# Artificial intelligence-based analysis of the spatial distribution of abnormal computed tomography patterns in SARS-CoV-2 pneumonia: association with disease severity

**DOI:** 10.1186/s12931-024-02673-w

**Published:** 2024-01-10

**Authors:** Yusuke Kataoka, Naoya Tanabe, Masahiro Shirata, Nobuyoshi Hamao, Issei Oi, Tomoki Maetani, Yusuke Shiraishi, Kentaro Hashimoto, Masatoshi Yamazoe, Hiroshi Shima, Hitomi Ajimizu, Tsuyoshi Oguma, Masahito Emura, Kazuo Endo, Yoshinori Hasegawa, Tadashi Mio, Tetsuhiro Shiota, Hiroaki Yasui, Hitoshi Nakaji, Michiko Tsuchiya, Keisuke Tomii, Toyohiro Hirai, Isao Ito

**Affiliations:** 1 Department of Respiratory Medicine, Graduate School of Medicine, Kyoto University, 54 Kawahara-Cho, Shogoin, Sakyo-Ku, Kyoto, 606-8507 Japan; 2 Department of Internal Medicine, Sugita Genpaku Memorial Obama Municipal Hospital, Obama, Japan; 3 Department of Respiratory Medicine, Kyoto City Hospital, Kyoto, Japan; 4 Department of Respiratory Medicine, Hyogo Prefectural Amagasaki General Medical Center, Amagasaki, Japan; 5 Department of Respiratory Medicine, Osaka Saiseikai Nakatsu Hospital, Osaka, Japan; 6 Division of Respiratory Medicine, Center for Respiratory Diseases, National Hospital Organization Kyoto Medical Center, Kyoto, Japan; 7 Division of Respiratory Medicine, Ako City Hospital, Ako, Japan; 8 Department of Internal Medicine, Horikawa Hospital, Kyoto, Japan; 9 Department of Respiratory Medicine, Toyooka Hospital, Toyooka, Japan; 10 Department of Respiratory Medicine, Rakuwakai Otowa Hospital, Kyoto, Japan; 11 Department of Respiratory Medicine, Kobe City Medical Center General Hospital, Kobe, Japan

**Keywords:** COVID-19, Pneumonia, Quantitative analysis, Ground glass opacity, Peripheral area, Central area

## Abstract

**Background:**

The substantial heterogeneity of clinical presentations in patients with severe acute respiratory syndrome coronavirus 2 (SARS-CoV-2) pneumonia still requires robust chest computed tomography analysis to identify high-risk patients. While extension of ground-glass opacity and consolidation from peripheral to central lung fields on chest computed tomography (CT) might be associated with severely ill conditions, quantification of the central-peripheral distribution of ground glass opacity and consolidation in assessments of SARS-CoV-2 pneumonia remains unestablished. This study aimed to examine whether the central-peripheral distributions of ground glass opacity and consolidation were associated with severe outcomes in patients with SARS-CoV-2 pneumonia independent of the whole-lung extents of these abnormal shadows.

**Methods:**

This multicenter retrospective cohort included hospitalized patients with SARS-CoV-2 pneumonia between January 2020 and August 2021. An artificial intelligence-based image analysis technology was used to segment abnormal shadows, including ground glass opacity and consolidation. The area ratio of ground glass opacity and consolidation to the whole lung (GGO%, CON%) and the ratio of ground glass opacity and consolidation areas in the central lungs to those in the peripheral lungs (GGO(C/P)) and (CON(C/P)) were automatically calculated. Severe outcome was defined as in-hospital death or requirement for endotracheal intubation.

**Results:**

Of 512 enrolled patients, the severe outcome was observed in 77 patients. GGO% and CON% were higher in patients with severe outcomes than in those without. Multivariable logistic models showed that GGO(C/P), but not CON(C/P), was associated with the severe outcome independent of age, sex, comorbidities, GGO%, and CON%.

**Conclusion:**

In addition to GGO% and CON% in the whole lung, the higher the ratio of ground glass opacity in the central regions to that in the peripheral regions was, the more severe the outcomes in patients with SARS-CoV-2 pneumonia were. The proposed method might be useful to reproducibly quantify the extension of ground glass opacity from peripheral to central lungs and to estimate prognosis.

**Supplementary Information:**

The online version contains supplementary material available at 10.1186/s12931-024-02673-w.

## Main text

### Introduction

The global pandemic of novel coronavirus disease 2019 (COVID-19) caused by severe acute respiratory syndrome coronavirus 2 (SARS-CoV-2) has generated an unprecedented health burden, with over 680 million cases and 6.9 million deaths worldwide as of June 22, 2023 [1]. Although many cases of COVID-19 remit spontaneously, some cases present a rapid deterioration from the onset of symptoms into severe illness [2–4]. The heterogeneity of clinical courses still requires the establishment of methods for identifying high-risk patients to appropriately use medical resources and improve outcomes.

Chest computed tomography (CT) is widely used for the diagnosis of COVID-19 and to predict the severity and prognosis of the disease. [5–11] Bilateral patchy ground-glass opacification/opacity (GGO) and consolidation (CON) are CT findings often observed in pneumonia caused by SARS-CoV-2. Initially, GGOs are localized in the subpleural or peripheral area, particularly in the lower lobes. Then, GGOs increase in size and extend to the central area with a crazy paving pattern and consolidation (CON) [10–12]. Studies have reported that GGOs, CON, air bronchograms, central lung involvement, and pleural effusion were associated with adverse clinical outcomes such as intensive care unit (ICU) admission or mortality [10, 13, 14]. Others have shown that semiquantitative visual scoring and quantification of GGO and CON may reflect disease severity and allow the prediction of adverse clinical outcomes [15–18]. However, quantitative analyses beyond visual inspection have yet to be fully established. No study has quantified the spatial distribution of abnormal CT patterns in central and peripheral lungs and calculated their central-to-peripheral ratio in relation to the severity and prognosis of COVID-19.

Recent advances in artificial intelligence (AI) have developed applications in various health care fields, including AI-based radiological diagnostic technology [19–21]. Deep learning is an AI method that employs artificial neural networks and allows for diagnostic assistance and prognosis prediction in patients with lung diseases, including interstitial lung disease and SARS-CoV-2 pneumonia [16, 22, 23]. Indeed, artificial intelligence-based quantitative CT image analysis software (AIQCT) was developed to automatically recognize and quantify abnormal parenchymal lesions on CT from patients with diffuse lung diseases [24].

It was hypothesized that the spatial distribution of GGO and CON would be associated with clinical outcomes in patients with SARS-CoV-2 pneumonia independent of the whole lung extents of GGO and CON. With the use of AIQCT, this study aimed to develop an automatic calculation system for the rate of lung parenchyma occupied by target CT patterns such as GGO and CON according to the distance from the pleura. We believe that the established system for quantification of the spatial distribution pattern of target radiological features could be the foundation of a clinically relevant platform for appropriate assessment of the evolved type of pneumonia caused by future strains of SARS-CoV-2 and even pneumonia caused by unknown pathogens.

## Study design and methods

### Patients

This retrospective study consecutively included adult patients with COVID-19 who underwent chest CT after admission at 9 tertiary hospitals in Japan between January 2020 and August 2021. The period corresponded to the first to fifth waves of COVID-19 in Japan [25]. B.1.1.284 and B.1.1.214 were the predominant lineages seen between March and October 2020 and between October 2020 and February 2021, respectively, in Japan. Then, B.1.1.7 (Alpha) and B.1.617 (Delta) prevailed between February and July 2021 and after August [26]. COVID-19 was diagnosed based on positive results of polymerase chain reaction and loop-mediated isothermal amplification assay for SARS-CoV-2. Severe outcomes were defined as either death or the need for endotracheal intubation. The exclusion criteria included (1) the absence of symptoms on admission, (2) missing predetermined clinical data such as age, sex, and comorbidities (chronic cardiovascular disease, chronic kidney disease, and diabetes), (3) the absence of pneumonia on CT by visual inspection, (4) age less than 16 years old, and (5) CT scan performed before the onset or more than 15 days after symptom onset. The research was conducted in compliance with the Declaration of Helsinki and was approved by the Ethics Committees of Kyoto University Hospital (approval No. R2407-1 and R2866-1). Written informed consent was waived due to the retrospective nature of the analysis.

### Data acquisition

We collected clinical data regarding age, sex, body mass index (BMI), smoking history, underlying diseases and laboratory data at admission, and outcome. Data were obtained from registries of participating hospitals.

### CT image analysis

**Fig. 1:**
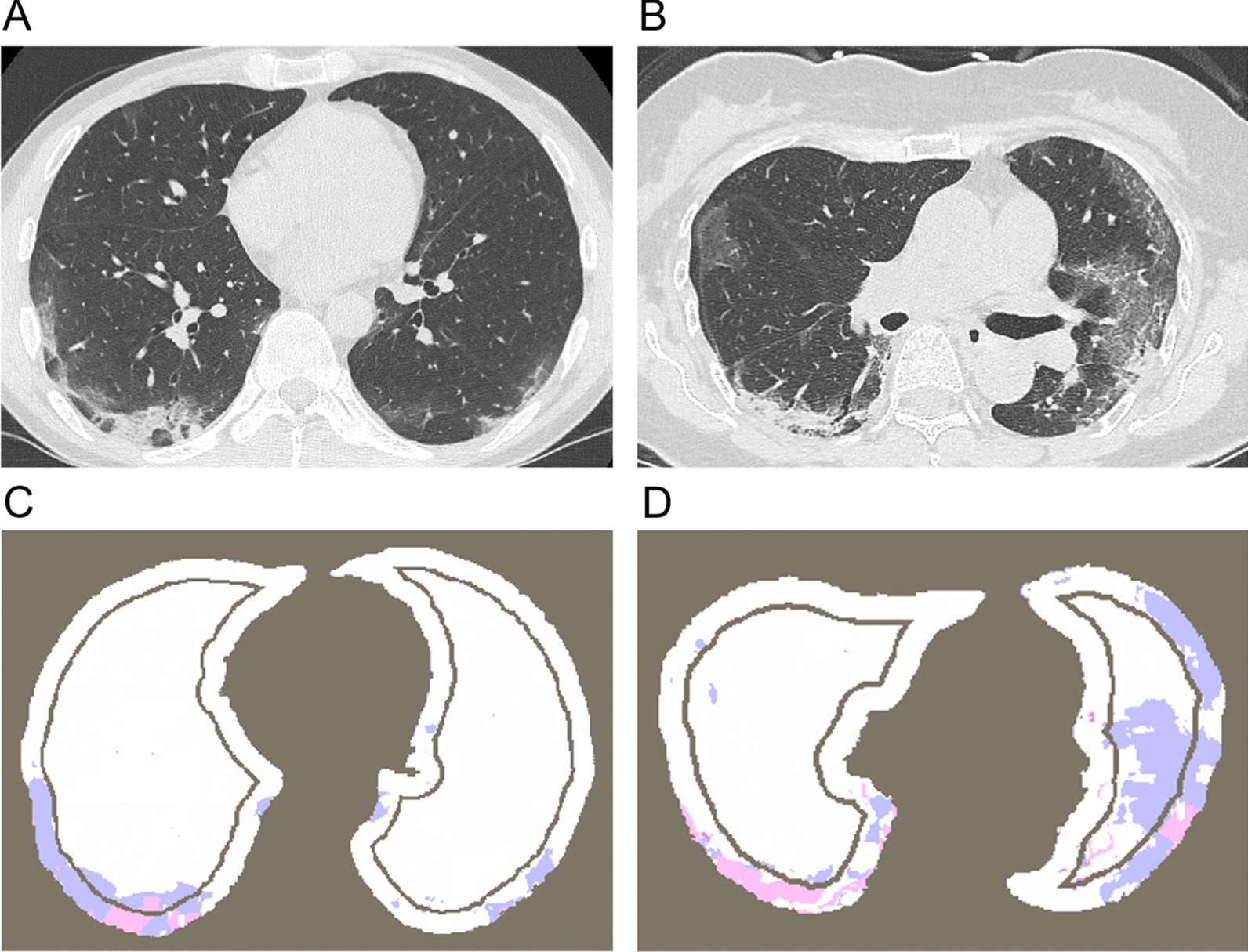
Representative images of SARS-CoV-2 pneumonia. **A** and **B** indicate two representative CT images of the nonsevere and severe cases, respectively. **C** and **D** are corresponding images after segmentation of lungs, ground-glass opacification/opacity (GGO) and consolidation (CON). The blue and pink colored areas indicate the area of GGO and CON respectively. In the nonsevere case (**A**, **C**), the percentages of whole, peripheral, and central lungs occupied by GGO (GGO%, GGO%(P), GGO%(C)) were 5.61%, 11.0%, and 2.22%, respectively. The percentage of whole, peripheral, and central lungs occupied by CON (CON%, CON%(P), CON%(C)) were 5.37%, 3.54%, and 6.52%, respectively. In the severe case (**B**, **D**), GGO%, GGO%(P), GGO%(C) were 9.68%, 10.8%, 8.31%, and CON%, CON%(P), CON%(C) were 12.1%, 15.0%, 8.70%

The study analyzed chest full-inspiratory CT obtained at the closest to the date of admission. Images were reconstructed with sharp kernels, and the slice thickness ranged from 0.5 to 5.0 mm. CT scans with a slice thickness greater than 5 mm or the presence of mediastinal emphysema, pneumothorax, pleural effusion, and lung cancer were excluded because of limited vertical consecutiveness of lung field images and the possibility that AIQCT could not appropriately identify the parenchymal structure around mediastinal emphysema, respectively. For CT analyses, the whole lung field was segmented based on AIQCT, and each pixel was assigned to either GGO, CON, reticulation (RET), honeycombing (HON), hyperlucent lung (LUC) mainly representing emphysema, other lung parenchyma, and outside lung using SYNAPSE VINCENT software (FUJIFILM, Tokyo, Japan). The software was initially validated using CT images of patients with idiopathic pulmonary fibrosis [24]. Then, the labeled images were exported as Digital Imaging and Communications in Medicine (DICOM) data and analyzed using a custom-made program based on Python modules. The segmented lung fields were divided into peripheral and central areas for each axial slice (see Fig. 1). The boundary was determined based on two methods: the distance from the pleura (length-based condition; 5, 10, 15, 20, 25 mm) and the prespecified percentage of the peripheral area to the total lung area for each axial slice (ratio-based condition; 25, 40, 60, 75% for the peripheral area). The latter could normalize intersubject variation in lung sizes. Furthermore, lungs were also vertically divided into 3 regions and GGO, CON, RET, HON, and LUC in each region for subanalyses (see Additional file 1).

The ratios of GGO, CON, RET, HON, and LUC were calculated individually as follows: the number of pixels in the specified regions in each slice was counted and added together from the apex to the base of the lung. Lungs were craniocaudally divided into 3 parts, including the apex, middle, and base regions occupying 10%, 80%, and 10% of the total lung volume, respectively. CT images in the apex and base regions were excluded from this analysis because the lung fields at the apex and base were too small to be analyzed in the central and peripheral areas separately. The total number of pixels obtained from the calculation was divided by the total number of pixels in the lung field area, which is defined as the “lesion ratio (GGO%, CON%, RET%, HON%, LUC%)” in this study. Additionally, to assess the combined effect of the parenchymal lesion ratio from both peripheral and central areas, the lesion ratio in the central area was divided by that in the peripheral area for GGO%, CON%, and RET%, which were defined as “GGO(C/P)”, “CON(C/P)”, or “RET(C/P)”, respectively.

### Statistical analysis

**S**tatistical analyses were performed using Python statistical software 3.10.11. Data are expressed as the mean ± standard deviation (SD) unless indicated. The details of the statistical analysis are described in the Supplemental Materials. Clinical and radiological data of the CT scans were compared between patients who developed severe outcomes (severe outcome group) and those who did not (nonsevere outcome group). Categorical and continuous variables were compared using a chi-square test and Wilcoxon's rank sum test, respectively. Correlations between two continuous variables were evaluated using the Pearson or Spearman correlation test. Three multivariable logistic regression models were constructed to explore factors associated with severe outcomes. The first model included age, sex, chronic heart disease, chronic kidney disease, diabetes mellitus and timing of CT acquisition as covariates (Model 1). The second model was constructed by adding GGO% and CON% to Model 1 (Model 2). The third model was constructed by adding the parenchymal ratio of abnormal shadows in the peripheral-central area (GGO(C/P), CON(C/P)) to Model 2 (Model 3). Moreover, for subanalysis including patients whose data of BMI and smoking status were available, multivariable models were constructed by adding BMI and smoking status to the variables used in the original models as covariates. A two-sided p value less than.05 was considered statistically significant.

## Results

### Patient characteristics

**Fig. 2:**
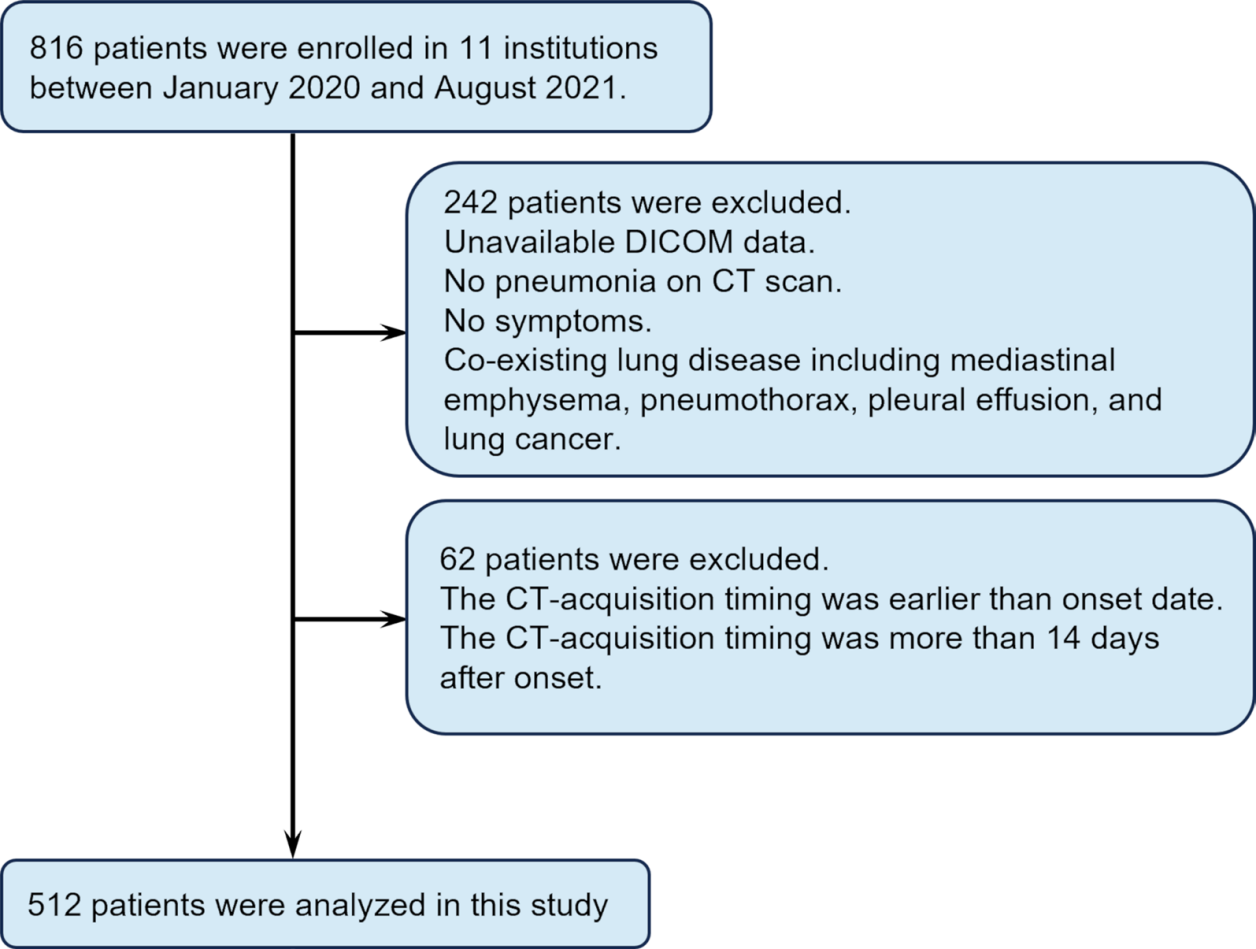
Patient flow chart. *DICOM*  digital imaging and communications in medicine. *AIQCT*  novel artificial intelligence-based quantitative CT image analysis software

Of 816 patients initially evaluated, 512 were included in the present analyses (Fig. 2). As shown in Table 1, severe outcomes were observed in 77 (15.0%) patients (death, n = 28, and endotracheal intubation, n = 61). The average time interval between symptom onset and CT acquisition was 5.9 (SD, 3.3) days. Age, and prevalence of underlying diseases such as chronic cardiovascular disease, chronic kidney disease, and diabetes mellitus differed between the severe and nonsevere outcome groups, while sex, BMI, smoking history, and the period between symptom onset and CT acquisition did not differ (Table 1).

**Table 1:** Patient characteristics

Characteristic	All cases	Nonsevere cases	Severe cases	*P* value
N	512	435	77	–
Age, y	60.6 ± 17.4	58.6 ± 17.5	71.9 ± 11.6	< 0.001
Sex, male	304 (59.4)	252 (57.9)	52 (67.5)	0.145
BMI	24.5 ± 4.30	24.5 ± 4.46	24.4 ± 3.47	0.687
Smoking status, current	65 (12.7)	59 (13.6)	6 (7.79)	0.223
Chronic heart disease	46 (8.98)	34 (7.82)	12 (15.6)	0.048
Chronic kidney disease	27 (5.27)	18 (4.14)	9 (11.7)	0.014
Diabetes	105 (20.5)	81 (18.6)	24 (31.2)	0.018
WBC [/µL]	5634 ± 2406	5429 ± 2072	6778 ± 3575	0.006
NLR [%]	31.1 ± 24.5	33.3 ± 24.8	18.6 ± 18.0	< 0.001
LDH [U/L]	307 ± 125	284 ± 101	436 ± 163	< 0.001
CRP [mg/dL]	5.88 ± 5.74	5.14 ± 5.10	10.0 ± 7.24	< 0.001
Cr [mg/dL]	1.01 ± 1.27	0.96 ± 1.05	1.33 ± 2.09	0.039
CT scan date, days	5.93 ± 3.29	5.92 ± 3.30	5.95(3.31)	0.967
Mortality	28(5.47)	0(0)	28(36.4)	–
Endotracheal intubation	61(11.9)	0(0)	61(79.2)	–

Data are presented as the means ± standard deviations or N (%). Data of body mass index (BMI), smoking status, white blood cell (WBC), neutrophil-to-lymphocyte ratio (NLR), lactate dehydrogenase (LDH), C-reactive protein (CRP), and creatinine (Cr) are available in 309, 449, 508, 493, 510, 509, and 508 patients, respectively. CT scan date = interval between symptom onset and CT acquisition

### Distribution pattern of lesion ratio

**Fig. 3:**
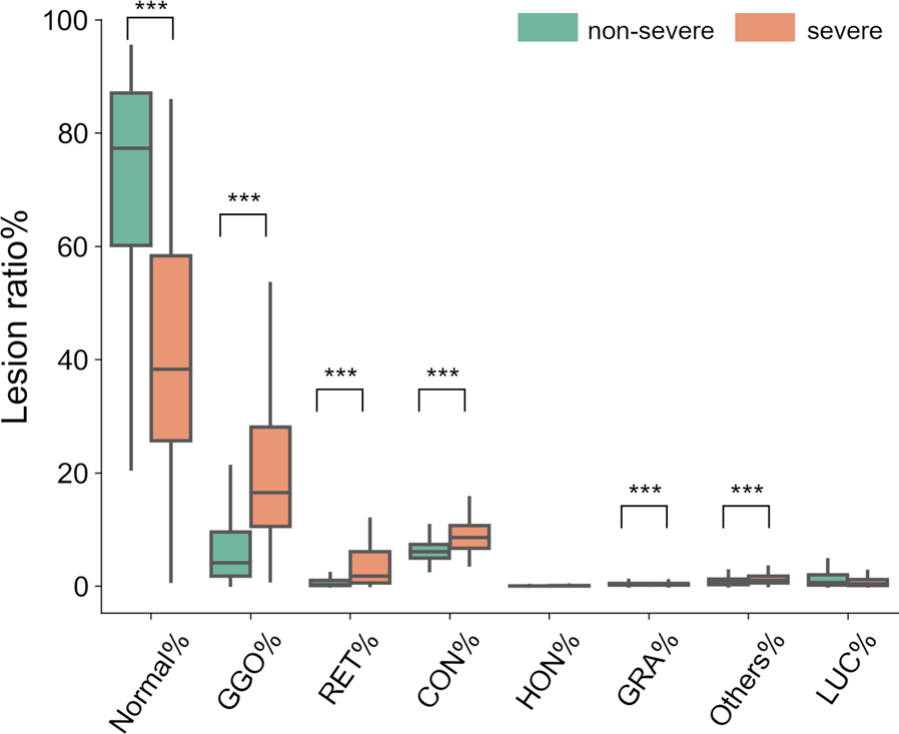
The distribution of parenchymal lesions in the whole lung area. The percentages of lungs occupied by each CT pattern was compared between nonsevere and severe cases. *Normal*  normal parenchymal, *GGO*  ground-glass opacification/opacity, *CON*  consolidation, *RET*  reticulation, *HON*  honeycomb lung, *GRA*  granular opacities, *LUC*  hyperlucent lung. *P* values were calculated by the Wilcoxon rank-sum test and Pearson correlation test [*P* < 0.05 (*); *P* < 0.01 (**); *P* < 0.001 (***)]

**Fig. 4:**
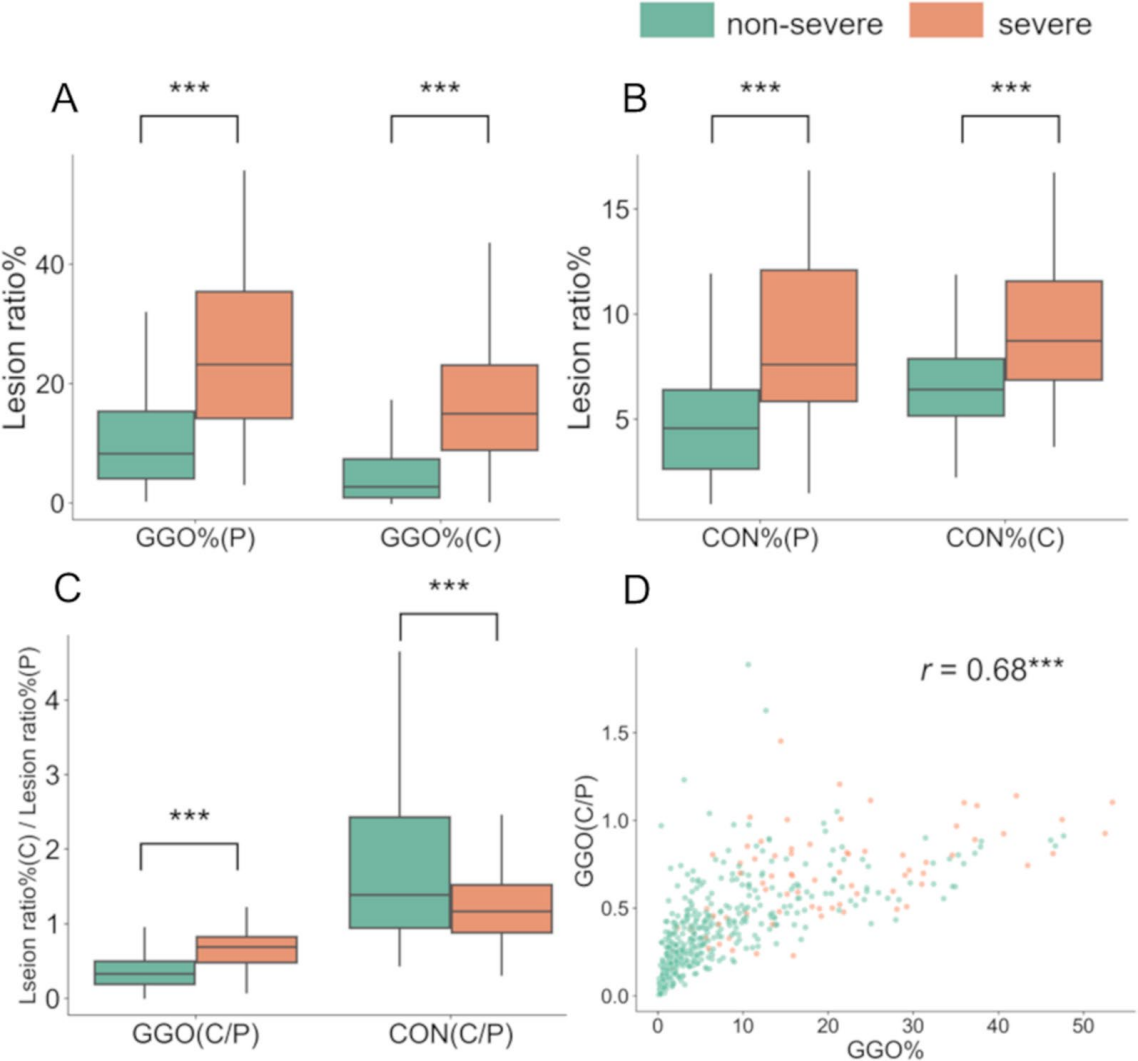
The distribution of the ratio of GGOs and CON in the peripheral or central lung region. **A** and **B** The percentage of lungs occupied by ground-glass opacification/opacity and consolidation were calculated in the peripheral and central regions (GGO%(P), GGO%(C), CON%(P), and CON%(C), respectively). **C** The central to peripheral ratio for GGO% and CON% were calculated (GGO(C/P) and CON(C/P)). **D** A correlation between GGO% and GGO(C/P). The boundary between the central and peripheral regions was set as 5 mm from the pleura. These variables were compared between patients with severe outcomes and those with nonsevere outcomes. *P* values were calculated by the Wilcoxon rank-sum test [*P* < 0.05 (*); *P* < 0.01 (**); *P* < 0.001 (***)]

A comparison of each finding between the two groups is shown in Fig. 3. GGO%, CON%, and RET% were higher, and normal parenchymal region % was lower in the severe outcome group than in the nonsevere outcome group (GGO%: *P* < 0.001, CON%: *P* < 0.001, RET%: *P* < 0.001, normal %: *P* < 0.001). In contrast, HON% and LUC% did not differ between them. Comparisons of GGO and CON with discrimination of the peripheral area and the central area, the boundary placed at 5 mm from the pleura, between the two groups are shown in Fig. 4. GGO% and CON% in both the peripheral and central areas were higher in the severe outcome group than in the nonsevere outcome group (GGO%(P): *P* < 0.001, GGO%(C): *P* < 0.001, CON%(P): *P* < 0.001, CON%(C): *P* < 0.001). Regarding the central-peripheral distribution, GGO(C/P) was higher, and CON(C/P) was lower in the severe outcome group than in the nonsevere outcome group. (GGO(C/P): *P* < 0.001, CON(C/P): *P* = 0.002) There was a positive correlation between GGO(C/P) and GGO% (r = 0.68, *P* < 0.001). Moreover, RET% in both the peripheral and central areas and RET(C/T) were also higher in the severe outcome group (RET%(P): *P* < 0.001, RET%(C): *P* < 0.001, RET(C/P): *P* < 0.001) (Additional file 1: Fig. S1).

### Clinical and CT imaging parameters associated with severe outcomes

As shown in Table 2, multivariable logistic regression models were constructed to evaluate the association of clinical and CT parameters with severe outcomes while placing the boundary from the pleura at 5 mm from the pleura. Among the clinical parameters, age and sex were associated with the development of severe outcomes (sex (male): odds ratio [OR], 1.99; 95% CI; 1.12, 3.53, *P* = 0.02, age: OR, 1.06; 95% CI; 1.04, 1.08, *P* < 0.001; Table 2 and Additional file 1: Fig. S2A, Model 1). Among the CT parameters, both higher GGO% and CON% in the whole lung area were associated with severe outcomes (GGO%: OR, 1.09; 95% CI; 1.06, 1.12, *P* < 0.001, CON%: OR, 1.12; 95% CI; 1.05, 1.20, *P* = 0.001) (Table 2 and Additional file 1: Fig. S2B, Model 2). Furthermore, GGO(C/P) but not CON(C/P) was associated with severe outcomes independent of GGO% and CON% in the whole lung area. (GGO(C/P): OR, 8.67; 95% CI; 2.30, 32.6, *P* = 0.001) (Table 2 and Additional file 1: Fig. S2C, Model 3). RET% in the whole lung area, but not RET(C/P), was also associated with severe outcomes (Additional file 1: Table S4, Model 2, Model 3). Additionally, the sooner the 1st CT was acquired from the date of hospitalization, the more severe the outcome was.

**Table 2:** Multivariable logistic regression models for factors associated with severe outcomes in SARS-CoV-2 pneumonia

Characteristics	Model 1		Model 2		Model 3	
	Odds ratio	*P v*alue	Odds ratio	*P* value	Odds ratio	*P* value
GGO(C/P)	–	–	–	–	8.67 (2.30–32.6)	0.001
CON(C/P)	–	–	–	–	0.89 (0.59–1.35)	0.58
GGO% whole lung	–	–	1.09 (1.06–1.12)	< 0.001	1.06 (1.02–1.09)	0.001
CON% whole lung	–	–	1.12 (1.05–1.20)	0.001	1.09 (1.01–1.17)	0.019
Sex (male)	1.99 (1.12–3.53)	0.02	2.79 (1.40–5.60)	0.004	2.80 (1.37–5.70)	0.005
Age	1.06 (1.04–1.08)	< 0.001	1.06 (1.03–1.08)	< 0.001	1.06 (1.03–1.08)	< 0.001
Chronic heart disease	1.17 (0.54–2.55)	0.69	0.855 (0.358–2.04)	0.72	0.74 (0.31–1.80)	0.51
Chronic kidney disease	1.47 (0.58–3.69)	0.41	1.71 (0.614–4.77)	0.30	2.03 (0.71–5.77)	0.19
Diabetes mellitus	1.24 (0.69–2.21)	0.47	0.74 (0.37–1.46)	0.38	0.80 (0.40–1.58)	0.52
CT-date	1.02 (0.95–1.01)	0.69	0.95 (0.87–1.04)	0.26	0.92 (0.84–1.01)	0.09

Values indicate Odds ratio (95% confidence interval). GGO% and CON% represent the percentage of lungs occupied by ground-glass opacification/opacity (GGO) and consolidation (CON), respectively. GGO(C/P) and CON(C/P) represent the ratios of GGO and CON areas in the central regions to those in peripheral regions, respectively. CT-date = interval between symptom onset and CT acquisition

Sensitivity analyses were performed in multivariable logistic regression models including GGO(C/P) and CON(C/P) based on different boundary conditions. The association of GGO(C/P) with the severe outcome independent of the whole lung GGO% and CON% was confirmed in models where the boundary distance from the pleura was set as 5 mm, 10 mm, 15 mm, and 20 mm but not 25 mm (Additional file 1: Table S3). The association was also confirmed even when defining the central-peripheral boundaries based on the ratio-based condition (cutoff value = 25%, 40%, 60%) (Additional file 1: Table S3). Additionally, when GGO(C/P) and CON(C/P) were quantified in the upper, middle, and lower lung areas separately, GGO(C/P) in the upper and lower lung areas, but not the middle areas, was associated with severe outcomes independent of the whole lung GGO% and CON% for all length-based conditions (distance from the pleura = 5 mm, 10 mm, 15 mm, 20 mm) (Additional file 1: Table S3). Moreover, in subanalyses including patients whose BMI and smoking status available, GGO(C/P) was significantly associated with severe outcomes (OR, 11.9; 95% CI; 1.38, 103, P = 0.02) independent of GGO% and CON% in the whole lung area (Additional file 1: Table S4).

Greater GGO%, CON%, GGO(C/P), and CON(C/P) on CT were associated with higher white blood cell (WBC), neutrophil-to-lymphocyte ratio (NLR), lactate dehydrogenase (LDH), and c-reactive protein (CRP) (Additional file 1: Fig. S3). GGO%, CON%, and GGO(C/P), but not CON(C/P), were higher in subjects with comorbidities of chronic heart disease and diabetes than those without (Additional file 1: Fig. S4).

## Discussion

This study localized GGO and CON on CT using AIQCT and showed that GGO% and CON% were higher and normal parenchymal region % was lower in patients with severe outcomes than in those without, whereas HON% and LUC% did not differ between them. Moreover, multivariable models showed that GGO (C/P), but not CON (C/P), was associated with a severe outcome independent of GGO% and CON% in the entire lungs. These findings suggest that the central-peripheral distribution of GGOs and CON may complement the overall severity of GGOs and CON in terms of prognostic evaluation and help perform more personalized management of patients with SARS-CoV-2 pneumonia.

The pathology of severe pneumonia due to SARS-CoV-2 infection is characterized by acute and organizing diffuse alveolar damage [27–30]. This may be radiologically identified as CON or a mixed pattern of GGO and CON on CT in critically ill cases with poor prognosis. Indeed, previous studies have shown that GGOs and CON in the whole lungs could predict disease deterioration and mortality [10], and the extension of GGOs along the subpleural area is associated with hypoxemia and SARS-CoV-2 viremia [31, 32]. Nonetheless, further improvement in radiological models for estimating the risk of poor prognosis is still warranted due to large clinical heterogeneity [33]. Therefore, this study is significant because it succeeded in automatically labeling multiple abnormal parenchymal lesions and quantifying each lesion in central and peripheral areas in relation to severe outcomes in patients with SARS-CoV-2 pneumonia.

This study determined the cutoff value of the distance from the pleura to define the boundary between central and peripheral areas and calculated GGO(C/P) and CON(C/P). GGO(C/P) was positively correlated with GGO% in the entire lung, but the correlation was not strong. Indeed, the multivariable models showed that both GGO(C/P) and GGO% in the entire lung were independently associated with severe outcomes. This extends previous studies showing that the extents of GGO or CON in the entire lung, as estimated by either semiquantitative scores or AI, including deep learning, were predictive of disease severity [16, 34].

CON%, but not CON(C/P), was associated with severe outcomes in this study. This seems inconsistent with a previous study showing that the extent of CON in the central region was associated with severe outcomes [14]. It is well known that GGO was replaced by CON [8, 10, 35]. In this study, the timing of chest CT in this study was relatively earlier (the mean value was 5 days from the in-hospitalization date), and in this early phase, the larger lesion ratio of GGO/CON was associated with the severity of the disease. Given that GGO(C/P) was associated with a severe outcome but CON(C/P) was not, it is conceivable that in the early phase, the more GGO extended to the central region, the more likely the lesion that would develop into diffuse alveolar damage was to distribute not only in the peripheral region but also more broadly. CON might not continuously extend from the pleura to the central region. It is also possible that CON may not originate from the pleura or subpleural region within 20 mm, as defined in our analysis. These factors might explain the observed lack of associations between CON(C/P) and the severe outcomes in this study.

Coexisting lung diseases, such as interstitial lung disease and COPD, could affect severe outcomes in patients with COVID-19 [36, 37]. While lung function tests and CT data before COVID-19 onset were unavailable for all patients, the AIQCT system allowed for segmenting fibrotic changes such as honeycomb and reticulation and emphysematous changes (hyperlucent lung). Consequently, there was no difference in HON% and LUC% between patients who developed severe outcomes and those who did not. Therefore, we estimate that the influences of the underlying lung comorbidities on the present findings would be minimal.

One would argue that the reticular shadow may affect the observed association between GGO and a severe outcome because GGO could change into linear or multifocal reticular shadows during the course of the disease [38]. However, we think that this possibility is less likely. In the multivariable logistic regression model including both the parenchymal ratio of reticulation in the peripheral-central lung area RET(C/P) and GGO(C/P), RET(C/P) was not associated with a severe outcome (Additional file 1: Table S4).

GGO%, CON%, GGO(C/P) and CON(C/P) were correlated with WBC, NLR, LDH, CRP, and comorbidities such as chronic heart disease and diabetes. Moreover, multivariable models in Table 2 showed that GGO(C/P) was associated with severe outcomes independent of the presence of chronic heart disease and kidney disease. While smoking history and higher BMI have been shown to be associated with poor outcomes in SARS-CoV-2 infection [39, 40], the subanalyses in this study showed that GGO(C/P) was associated with severe outcomes independent of BMI and smoking history. Taken together, we postulate that GGO(C/P) could reflect previously-reported factors for severe pneumonia [41, 42], but still have independent impacts on severe outcomes in patients with SARS-CoV-2 pneumonia.

The data in this study did not include cases with omicron infection or those with pneumonia induced by other pathogens. Since omicron is currently a common mutant and the severe cases are less frequent in omicron infection than in previous forms of mutant infection, CT patterns in omicron infection might be different from those analyzed in this study. Because of lack of data, we could not examine whether the present findings can be directly applied to CT in cases with current SARS-CoV-2 mutant infection. Nonetheless, we believe our proposed method is clinically relevant. The method is based on a combination of AI-based segmentation of various abnormal shadow patterns and allows automatic calculation of spatial distribution patterns for specific target shadows, not limited to GGO and consolidation. Therefore, it can be readily tailored to apply for cases with pneumonia induced by various pathogens. With this method, further studies should be performed to explore whether similar findings are observed in cases with current SARS-CoV-2 mutant infection and to define specific CT features of SARS-CoV-2 pneumonia by comparing CT findings of SARS-CoV-2 infection to those induced by other pathogens.

The strength of our study is the use of large data collected from more than 500 patients with SARS-CoV-2 pneumonia from multiple institutions in Japan. Second, the use of AIQCT enabled the identification of interstitial fibrosis and emphysema as reticular and honeycomb shadows and hyperlucent regions and the estimation of the effect of coexisting lung diseases, such as interstitial lung disease and emphysema, on severe outcomes. However, this study has limitations. First, the CT protocols, including the timing of acquisition, slice thickness, reconstruction kernel, and scanner manufacturer, varied among institutions. Regarding the timing of CT acquisition, the multivariable regression models showed that the timing of CT was associated with severe outcomes for some boundary conditions. The imbalance in CT slice thickness might have affected the results, although the data analyses were performed using our dataset, which included CT scans with a thickness of more than 2 mm, thicker compared to a previous study [16]. Third, the training CT images for generating the AIQCT system used in this study did not include those with pleural effusion, pneumothorax, and pulmonary lung tumors [24]. Thus, these radiological findings may not be segmented correctly with AIQCT, and patients with these CT findings were excluded from the present analyses. Fourth, this study examined the associations of the CT findings with short-term outcomes, but not long-term outcomes including long COVID, which is an ongoing problem [43]. Further studies are needed to test whether the proposed CT metrics could be associated with long COVID using long-term longitudinal data. Finally, this study included only Japanese patients. Further studies are needed to determine the applicability of the present findings to other racial populations.

## Conclusion

In summary, this study showed that in addition to the whole lung extents of GGOs and consolidation, a higher ratio of GGOs in the central regions to those in the peripheral regions was associated with severe outcomes in patients with severe SARS-CoV-2 pneumonia. These findings encompass basic techniques that can be applied for better management of SARS-CoV-2 pneumonia as well as pneumonia caused by pandemic viruses that may occur in the future.

### Supplementary Information

**Additional file 1: Table S1.** Cutoff values to define the boundary between peripheral and central lung region. **Table S2.** Sensitivity analysis of multivariable logistic regression models with GGO(C/P). **Table S3.** Multivariable logistic regression models for associations of clinical and CT parameters with severe outcomes. **Table S4.** Sub-analysis of multivariable logistic regression models for factors associated with severe outcomes. **Figure S1.** The distribution of the ratio of reticulation in the peripheral or central lung region. **Figure S2.** Multivariable logistic regression models for factors associated with severe outcomes in SARS-CoV-2 pneumonia. **Figure S3.** The correlation of CT measurements with clinical measurements. **Figure S4.** The correlation of CT measurements with comorbidities.

## Data Availability

The datasets used and/or analyzed during the current study are available from the corresponding author on reasonable request.

## Abbreviations

COVID-19: Novel coronavirus disease 2019
SARS-CoV-2: Severe acute respiratory syndrome coronavirus 2
CT: Computed tomography
GGO: Ground-glass opacification/opacity
CON: Consolidation
ICU: Intensive care unit
AI: Artificial intelligence
AIQCT: Artificial intelligence-based quantitative CT image analysis software
RET: Reticulation
HON: Honeycombing
LUC: Hyperlucent lung
GGO%: The parenchymal ratio of ground-glass opacification/opacity in the whole lung area
CON%: The parenchymal ratio of consolidation in the whole lung area
RET%: The parenchymal ratio of reticulation in the whole lung area
GGO(C/P): The central to peripheral ratio for GGO%
CON(C/P): The central to peripheral ratio for CON%
RET(C/P): The central to peripheral ratio for RET%
WBC: White blood cell
NLR: Neutrophil-to-lymphocyte ratio
LDH: Lactate dehydrogenase
CRP: C-reactive protein
Cr: Creatinine
DICOM: Digital Imaging and Communications in Medicine
SD: Standard deviation
BMI: Body mass index
