# Supplementary material for: Artificial intelligence-based analysis of the spatial distribution of abnormal computed tomography patterns in SARS-CoV-2 pneumonia: association with disease severity
Source: Respir Res. 2024 Jan 10;25:24. doi: 10.1186/s12931-024-02673-w (PMC10777587; doi:10.1186/s12931-024-02673-w)
Supplement: Supplementary file 1 — Additional file 1: Table S1. Cutoff values to define the boundary between peripheral and central lung region. Table S2. Sensitivity analysis of multivariable logistic regression models with GGO(C/P). Table S3. Multivariable logistic regression models for associations of clinical and CT parameters with severe outcomes. Table S4. Sub-analysis of multivariable logistic regression models for factors associated with severe outcomes. Figure S1. The distribution of the ratio of reticulation in the peripheral or central lung region. Figure S2. Multivariable logistic regression models for factors associated with severe outcomes in SARS-CoV-2 pneumonia. Figure S3. The correlation of CT measurements with clinical measurements. Figure S4. The correlation of CT measurements with comorbidities. [file 12931_2024_2673_MOESM1_ESM.docx]

**Additional materials**

**Artificial intelligence-based analysis of the spatial distribution of abnormal computed tomography patterns in SARS-CoV-2 pneumonia: association with disease severity**

**Contents**

**1. Methods**

- CT acquisition and visual CT analysis.

**2. Tables**

- Table S1. Cutoff values to define the boundary between peripheral and central lung region.
- Table S2. Sensitivity analysis of multivariable logistic regression models with GGO(C/P).
- Table S3. Multivariable logistic regression models for associations of clinical and CT parameters with severe outcomes.
- Table S4. Sub-analysis of multivariable logistic regression models for factors associated with severe outcomes.

**3. Figures**

- Figure S1. The distribution of the ratio of reticulation in the peripheral or central lung region.
- Figure S2. Multivariable logistic regression models for factors associated with severe outcomes in SARS-CoV-2 pneumonia.
- Figure S3. The correlation of CT measurements with clinical measurements.
- Figure S4. The correlation of CT measurements with comorbidities.

1. **Methods**

**CT acquisition and visual CT analysis**

The study used CT scans acquired after symptom onset of COVID-19. CT images were reconstructed using sharp kernels with slice thicknesses ranging from 0.5 to 5.0 mm. Each abnormal parenchymal lesion was segmented and labeled by novel artificial intelligence-based quantitative CT image analysis software (AIQCT) ^1^. Ground-glass opacities (GGOs), reticulation, consolidation (CON), honeycomb lung (HON), granular shadow (GRA), hyperlucent lung (LUC), normal, and others were included in this study. Bronchi and vessels within the lung were excluded from the analysis.

The labeled images were exported from AIQCT software as DICOM format and used for subsequent processing with an original custom program based on Python modules. To calculate the whole-lung extent and the central-peripheral ratio of each radiological pattern, the whole lung field was first segmented by defining the lung parenchyma as pixels assigned to either GGO, reticulation, CON, HON, GRA, LUC, normal, or others. The segmented lung fields were split into peripheral and central lung fields via a boundary line. The boundary line on the axial plane for either the length-based condition or the ratio-based condition was defined based on the distance value from the pleura. The calculation of the distance value from the pleura was conducted using the Python module DistanceMap. In the ratio-based condition, repetitive calculation for the percentage of the peripheral area to the total lung area (peripheral%) was conducted for each axial slice until the value was close enough to the prespecified cutoff value. The calculation procedure was as follows: 1) The initial value of the boundary line started from 0.1 mm from the pleura. 2) The peripheral area and central area were defined based on the boundary line, and peripheral% was estimated. 3) If the peripheral% was less than the preset cutoff value, a new value for the boundary line was set by adding 0.1 mm to the previous value for the boundary line, and new peripheral% was estimated again. 4) Procedure 3) was repeated until peripheral% surpassed the cutoff value. e-Table 1 summarizes the peripheral % and distance value from the pleura corresponding to each cutoff value.

Then, for vertical multilung region analysis, those segmented areas were also split into 3 regions from the apex to the base of the lung. In the multilung region analysis, the height of each vertically split lung region was set to be equal. From the apex to the base of the lung, 3 lung regions were referred to as the upper, middle, and lower lung regions.

**reference**

1. Handa T, Tanizawa K, Oguma T, et al. Novel Artificial Intelligence-based Technology for Chest Computed Tomography Analysis of Idiopathic Pulmonary Fibrosis. *Ann Am Thorac Soc.* 2022;19(3):399-406.

**2. Tables**

**Table S1. Cutoff values to define the boundary between peripheral and central lung region.**

| cut off value [%] | mean ratio of peripheral area [%] | median value of distance from pleural [mm] |
| --- | --- | --- |
| 25 | 25.6 (0.77) | 5.80 (4.80-6.40) |
| 40 | 40.4 (0.60) | 9.60 (7.80-10.50) |
| 60 | 60.3 (0.43) | 15.4 (12.7-17.0) |
| 75 | 75.2 (0.27) | 20.7 (17.4-22.9) |

Numbers in parentheses in mean ratio of peripheral area [%] are standard deviation. Numbers in parentheses in median value of distance from pleura [mm] are quartile range.

Cutoff value = the prespecified percentage of the peripheral area to the total lung area

**Table S2.** **Sensitivity analysis of multivariable logistic regression models with GGO(C/P).**

| Distance  from pleural[mm] | *P* Value | Odds Ratio | *P* Value | Odds Ratio | *P* Value | Odds Ratio | *P* values | Odds Ratio |
| --- | --- | --- | --- | --- | --- | --- | --- | --- |
|  | whole lung area | | upper lung area | | middle lung area | | lower lung area | |
| 5 | .001 | 8.67(2.30-32.6) | .001 | 5.05(1.91-13.3) | .092 | 2.29(0.874-5.97) | .002 | 6.60(2.03-21.5) |
| 10 | .002 | 6.77(2.06-22.3) | .001 | 4.51(1.90-10.7) | .107 | 2.16(0.847-5.49) | .003 | 4.72(1.72-13.0) |
| 15 | .005 | 4.31(1.55-11.9) | .002 | 3.62(1.61-8.14) | .149 | 1.76(0.815-3.81) | .009 | 3.09(1.32-7.24) |
| 20 | .017 | 2.83(1.20-6.66) | .006 | 2.83(1.34-5.98) | .209 | 1.50(0.797-2.84) | .020 | 2.30(1.14-4.63) |
| 25 | .072 | 1.86(0.947-3.66) | .006 | 2.57(1.32-5.02) | .368 | 1.27(0.755-2.14) | .153 | 1.53(0.854-2.74) |
| Cutoff value [%] | *P* Value | Odds Ratio | *P* Value | Odds Ratio | *P* Value | Odds Ratio | *P* values | Odds Ratio |
|  | whole lung area | | upper lung area | | middle lung area | | lower lung area | |
| 25 | .002 | 9.02(2.28-35.6) | .001 | 5.16(1.94-13.7) | .114 | 2.14(0.833-5.50) | .009 | 6.69(1.61-27.8) |
| 40 | .001 | 7.85(2.29-27.0) | .001 | 4.56(1.89-11.0) | .111 | 2.02(0.852-4.77) | .005 | 6.29(1.73-22.8) |
| 60 | .004 | 4.21(1.59-11.1) | .002 | 3.68(1.61-8.41) | .160 | 1.59(0.833-3.04) | .007 | 4.12(1.47-11.6) |
| 75 | .027 | 2.15(1.09-4.23) | .005 | 2.93(1.38-6.22) | .292 | 1.28(0.81-2.03) | .025 | 2.39(1.11-5.12) |

Numbers in parentheses are 95% CIs.

GGO(C/P) = the ratio of ground-glass opacification/opacity in the central-peripheral lung area.

**Table S3.** **Multivariable logistic regression models for associations of clinical and CT parameters with severe outcomes.**

| Characteristics | Model 2 | | Model 3 | |
| --- | --- | --- | --- | --- |
|  | Odds Ratio | *P* Value | Odds Ratio | *P* Value |
| GGO(C/P) | - | - | 18.9 (4.28-83.5) | <.001 |
| RET(C/P) | - | - | 0.46 (0.152-1.38) | .16 |
| GGO% | 1.10 (1.07-1.13) | <.001 | 1.06 (1.03-1.10) | <.001 |
| RET% | 1.09 (1.01-1.18) | .02 | 1.10 (1.00-1.20) | .04 |
| sex | 2.67 (1.34-5.30) | .005 | 2.83 (1.38-5.83) | .005 |
| age | 1.05 (1.02-1.07) | <.001 | 1.06 (1.03-1.08) | <.001 |
| CHD | 0.69 (0.27-1.77) | .44 | 0.60 (0.23-1.57) | .30 |
| CKD | 1.70 (0.60-4.78) | .32 | 1.92 (0.66-5.63) | .23 |
| Diabetes mellitus | 0.77 (0.39-1.49) | .43 | 0.79 (0.40-1.57) | .51 |
| CT-date | 0.94 (0.86-1.03) | .16 | 0.90 (0.82-0.99) | .03 |

The numbers in parentheses are 95% CIs. The boundary distance from the pleural was set as 5 mm. GGO% = the parenchymal ratio of ground-glass opacification/opacity in the whole lung area, RET% = the parenchymal ratio of reticulation in the whole lung area, GGO(C/P) = the ratio of ground-glass opacification/opacity in the central-peripheral lung area, RET(C/P) = the ratio of reticulation in the central-peripheral lung area, CHD = chronic heart disease, CKD = chronic kidney disease, CT-date = interval between symptom onset and CT acquisition.

**Table S4. Sub-analysis of multivariable logistic regression models for factors associated with severe outcomes in SARS-CoV-2 pneumonia.**

| Characteristics | Model 1 | | Model 2 | | Model 3 | |
| --- | --- | --- | --- | --- | --- | --- |
|  | Odds Ratio | *P v*alue | Odds Ratio | *P* value | Odds Ratio | *P* Value |
| GGO(C/P) | - | - | - | - | 11.9 (1.38-103) | .03 |
| CON(C/P) | - | - | - | - | 0.79 (0.45-1.39) | .41 |
| GGO% whole lung | - | - | 1.08 (1.04-1.12) | <.001 | 1.04 (1.00-1.09) | .08 |
| CON% whole lung | - | - | 1.10 (1.01-1.21) | .03 | 1.06 (0.96-1.16) | .24 |
| Sex (male) | 1.35 (0.56-3.24) | .51 | 1.23 (0.47-3.19) | .68 | 1.27 (0.49-3.31) | .63 |
| Age | 1.05 (1.02-1.07) | .002 | 1.05 (1.01-1.08) | .005 | 1.05 (1.01-1.08) | .006 |
| BMI | 1.04 (0.95-1.13) | .40 | 1.00 (0.92-1.10) | .95 | 1.01 (0.92-1.11) | .80 |
| Smoking | 1.51 (0.88-2.60) | .14 | 1.57 (0.86-2.89) | .14 | 1.63 (0.87-3.02) | .13 |
| Chronic heart disease | 2.00 (0.80-5.01) | .14 | 1.73 (0.63-4.76) | .29 | 1.40 (0.49-4.04) | .53 |
| Chronic kidney disease | 2.26 (0.76-6.72) | .14 | 2.66 (0.77-9.20) | .12 | 3.30 (0.93-11.8) | .07 |
| Diabetes mellitus | 0.76 (0.33-1.73) | .51 | 0.48 (0.18-1.25) | .13 | 0.50 (0.19-1.32) | .16 |
| CT-date | 1.07 (0.97-1.18) | .20 | 1.02 (0.91-1.14) | .73 | 0.97 (0.86-1.10) | .66 |
| Values indicate 95% confidence interval. The total number of the datasets is 273. GGO% and CON% represent the percentage of lungs occupied by ground-glass opacification/opacity (GGO) and consolidation (CON), respectively. GGO(C/P) and CON(C/P) represent the ratios of GGO and CON areas in the central regions to those in peripheral regions, respectively. CT-date = interval between symptom onset and CT acquisition. | | | | | | |

**3. Figures**

**Figure S1.** **The distribution of the ratio of reticulation in the peripheral or central lung region.**

**
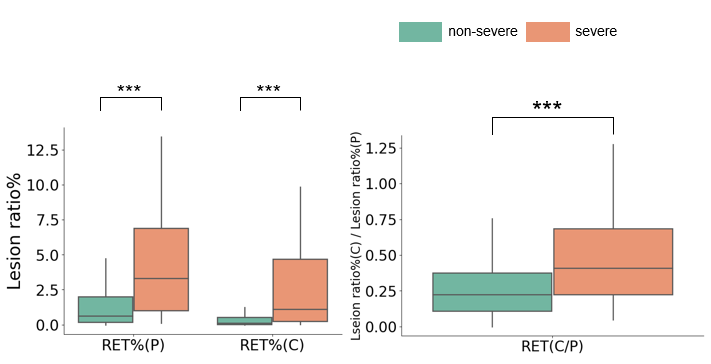
**

RET% = the parenchymal ratio of reticulation in the whole lung area, RET(C/P) = the ratio of reticulation in the central-peripheral lung area. *P* values were calculated by the Wilcoxon rank-sum test [*P* <0.05 (*); *P* <0.01 (**); *P* <0.001 (***)].

**Figure S2. Multivariable logistic regression models for factors associated with severe outcomes in SARS-CoV-2 pneumonia.**


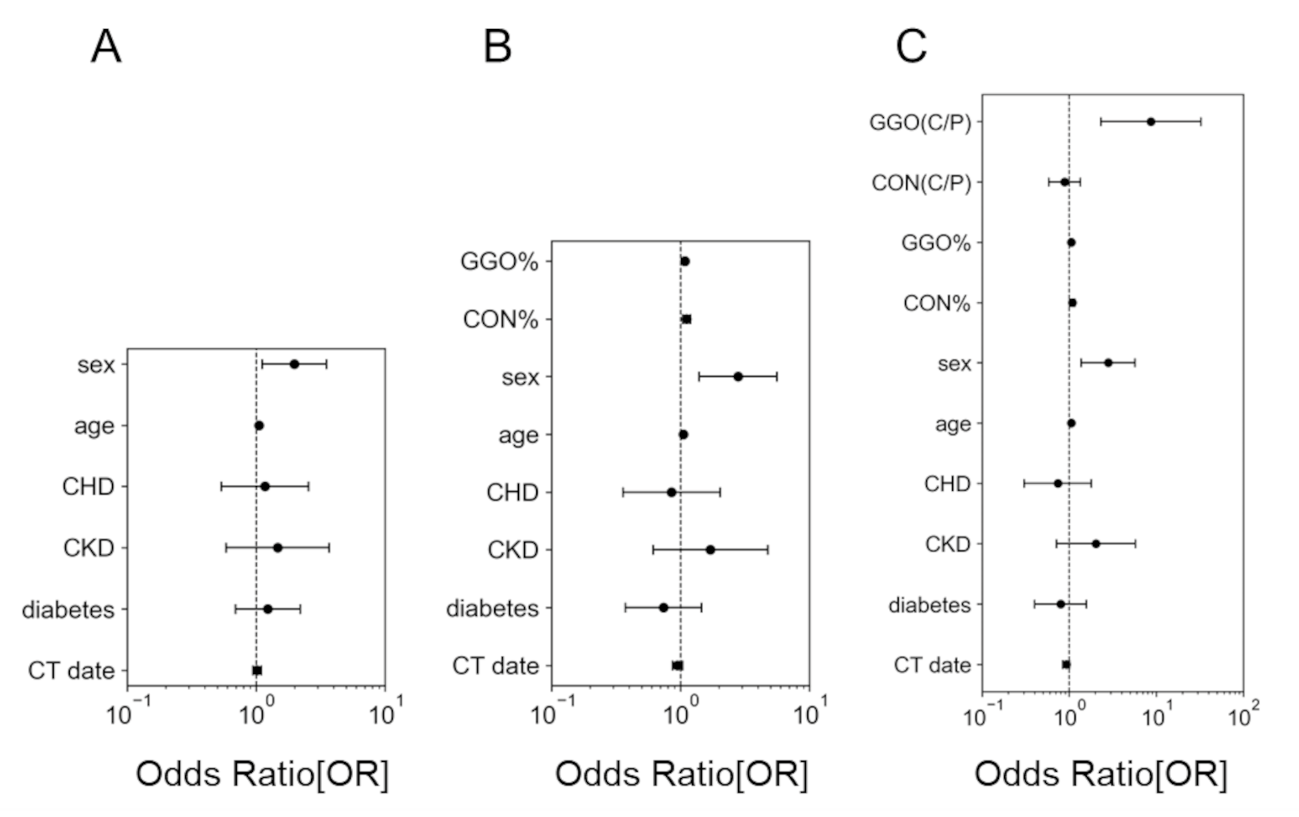


The basic multivariable model (Model A) was constructed by including clinical parameters. Model B was constructed by adding the percentage ratios of lungs occupied by ground-glass opacification/opacity (GGO%) and consolidation (CON%) to Model A. Model C was constructed by adding the ratios of ground-glass opacification/opacity and consolidation in the central regions to those in the peripheral regions (GGO(C/P) and CON(C/P)). The dots and bars indicate odds ratios (ORs) with 95% confidence intervals. CHD = chronic heart disease, CKD = chronic kidney disease, CT-date = interval between symptom onset and CT acquisition.

**Figure S3. The correlation of CT measurements with clinical measurements.**


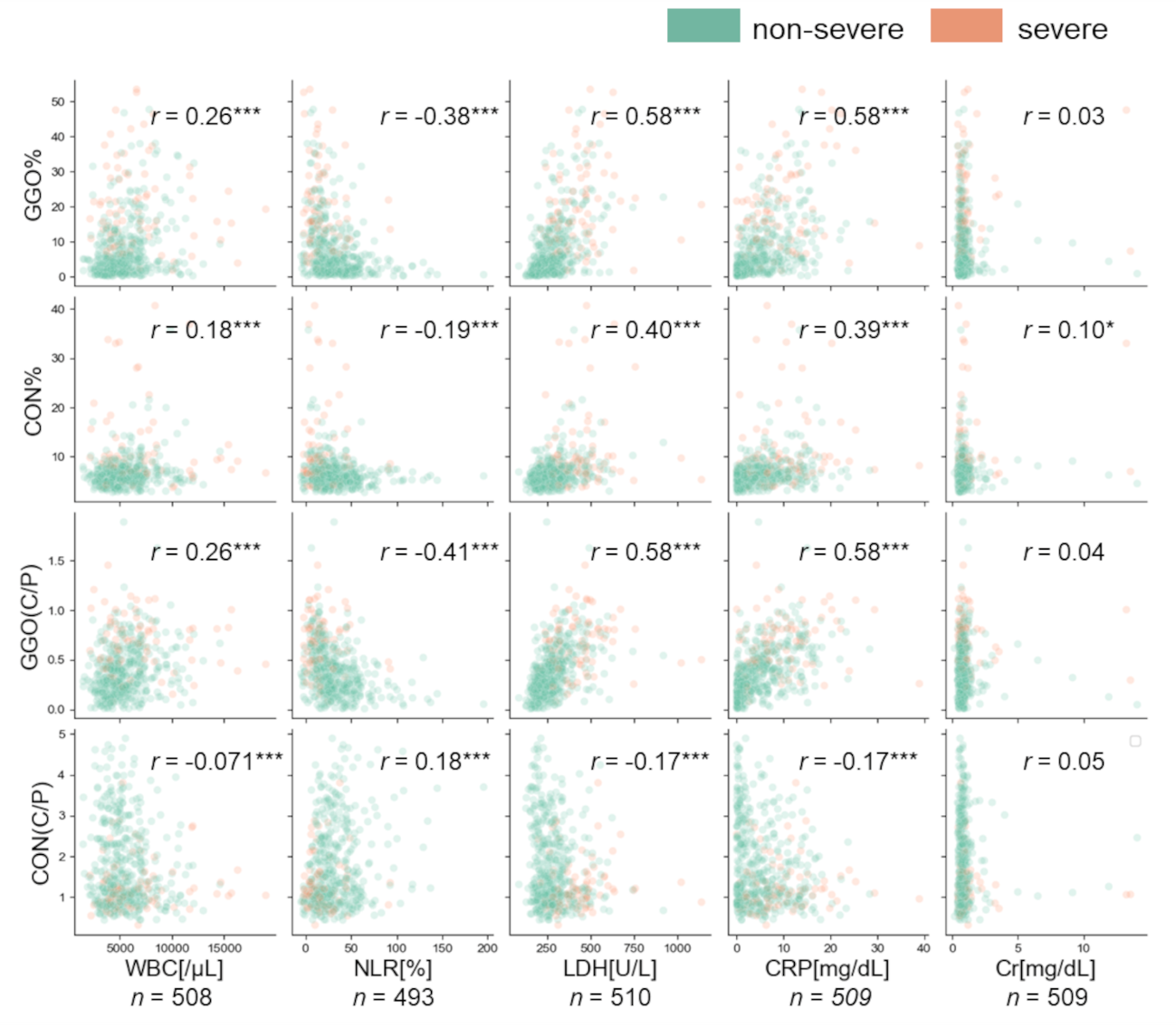


The correlation was calculated by Spearman's correlation coefficients method. GGO% = the parenchymal ratio of ground-glass opacification/opacity in the whole lung area, CON% = the parenchymal ratio of consolidation in the whole lung area, GGO(C/P) = the ratio of ground-glass opacification/opacity in the central-peripheral lung area, CON(C/P) = the ratio of consolidation in the central-peripheral lung area, WBC = White blood count, Lym/Neu = the ratio of the number of lymphocytes to that of neutrophils, LDH = Lactate dehydrogenase, CRP = C-reactive protein, Cr = Creatinine. *P* values were calculated by the Wilcoxon rank-sum test [*P* <0.05 (*); *P* <0.01 (**); *P* <0.001 (***)].

**Figure S4. The correlation of CT measurements with comorbidities.**


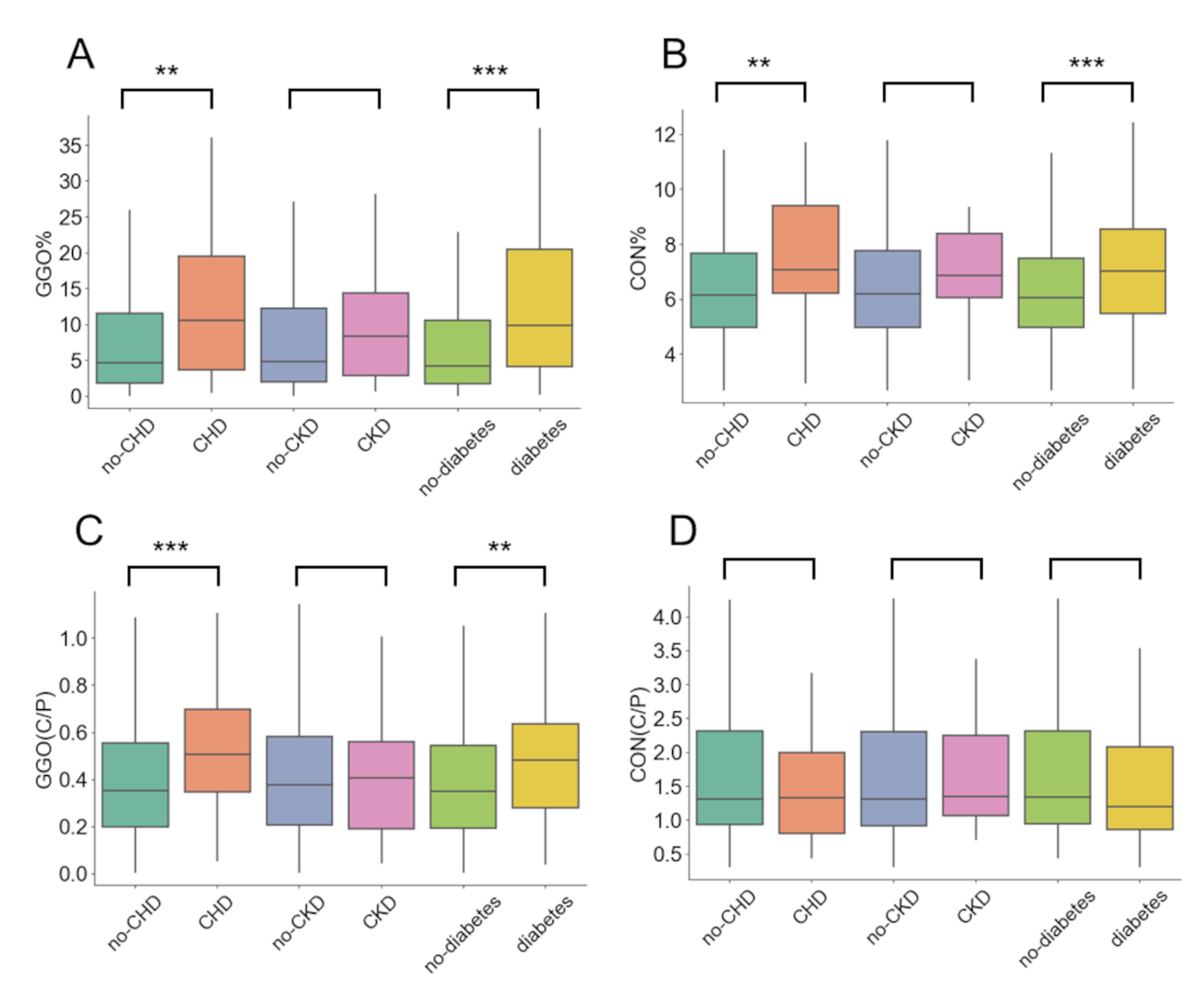


A and B. The percentage of lungs occupied by ground-glass opacification/opacity and consolidation (GGO% and CON%) were calculated in subjects with or without comorbidities including chronic heart disease (CHD), chronic kidney disease (CKD) and diabetes mellitus (diabetes). C and D. The central to peripheral ratio for GGO% and CON% (GGO(C/P) and CON(C/P)) were calculated in subjects with or without comorbidities including CHD, CKD and diabetes. P values were calculated by the Wilcoxon rank-sum test [*P* <0.05 (*); *P* <0.01 (**); *P* <0.001 (***)].
